# Aorta Fluorescence Imaging by Using Confocal Microscopy

**DOI:** 10.5402/2011/215627

**Published:** 2011-07-09

**Authors:** Chun-Yang Wang, Jui-che Tsai, Ching-Cheng Chuang, Yao-Sheng Hsieh, Chia-Wei Sun

**Affiliations:** ^1^Biophotonics Interdisciplinary Research Center and Institute of Biophotonics, National Yang-Ming University, Taipei 11221, Taiwan; ^2^Department of Photonics, National Chiao Tung University, Hsinchu 30010, Taiwan; ^3^Graduate Institute of Photonics and Optoelectronics and Department of Electrical Engineering, National Taiwan University, Taipei 10617, Taiwan; ^4^Institute of Biomedical Engineering, National Taiwan University, Taipei 10051, Taiwan

## Abstract

The activated leukocyte attacked the vascular endothelium and the associated increase in VEcadherin number was observed in experiments. The confocal microscopic system with a prism-based wavelength filter was used for multiwavelength fluorescence measurement. Multiwavelength fluorescence imaging based on the VEcadherin within the aorta segment of a rat was achieved. The confocal microscopic system capable of fluorescence detection of cardiovascular tissue is a useful tool for measuring the biological properties in clinical applications.

## 1. Introduction

In recent years, the confocal microscopic technique has had an important role in biology and medicine, especially for fluorescence imaging of tissues [1–6]. Compared with the conventional optical microscopy, the confocal microscopy provides more advantages, including controllable depth of field, elimination of out-of-focus information, and the capability to collect serial optical sections from thick specimens. Recently, fibered confocal fluorescence microscopy was combined in endoscopic imaging system [7–9]. Confocal endomicroscope aims at providing to the clinician optical biopsies, that is, *in vivo* microscopic imaging of a living tissue. Such systems have been successfully applied to the *in vivo* explorations of the human skin, cervix, and oral cavity, as well as to the endomicroscopic imaging of the gastric and colonic mucosa and biliary tract. Lately, the microscopic imaging was also achieved in the proximal and distal respiratory systems [10].

Atherosclerosis may occur in the arteries of the brain, heart, kidneys, vital organs, arms, and legs. It is the major cause for myocardial infarction, stroke, and peripheral vascular diseases. It is also the leading cause for illness and deaths in the United States and other developed countries [11]. Atherosclerosis has been studied extensively with various animal models [12]. The degree of low-density lipoprotein- (LDL-) induced leukocyte adhesion to endothelium is considered to be an important factor of developing atherosclerosis, and one of the earliest stages of atherogenesis is endothelial dysfunction [13]. In animal study using rats, the aorta endothelium has a relatively small thickness of ~100 *μ*m, which makes it difficult to obtain images under a conventional microscope. The confocal fluorescence microscope is a suitable alternative for acquiring sectioned images. It has observed LDL-induced leukocyte adhesion to endothelium.

Simultaneous multiwavelength fluorescence screening, for example, in observing certain proteins labeled with different fluorescent dyes [14–16], has attracted increasing attention [17]. Multiple excitation wavelengths are emitted from various lasers. The multiwavelength fluorescence signals can be measured by detectors with optical filters. In a conventional confocal fluorescence microscope, normally a filter wheel with chosen wavelength ranges was used [18, 19]. However, with the choice of dyes widening, the design needs be modified for multiwavelength measurement as detection based on rotating the filter wheel for different wavelengths with longer time-consuming and higher cost. Therefore, an adjustable design for optical filters is needed to involve the wavelength range of fluorescence [20–22].

In this paper, a confocal spectral microscope was used to measure the multiwavelength fluorescence images from the cut-open rat aorta. Both auto- and extra-fluorescence detections have been achieved in experiments. Controllable prism-based wavelength filters are incorporated into our microscopic system to obtain the multiwavelength fluorescence images [23]. The activated leukocyte attacks the vascular endothelium and the associated increase in VEcadherin number is observed in the experiments. 

## 2. Experiments

**Figure 1:**
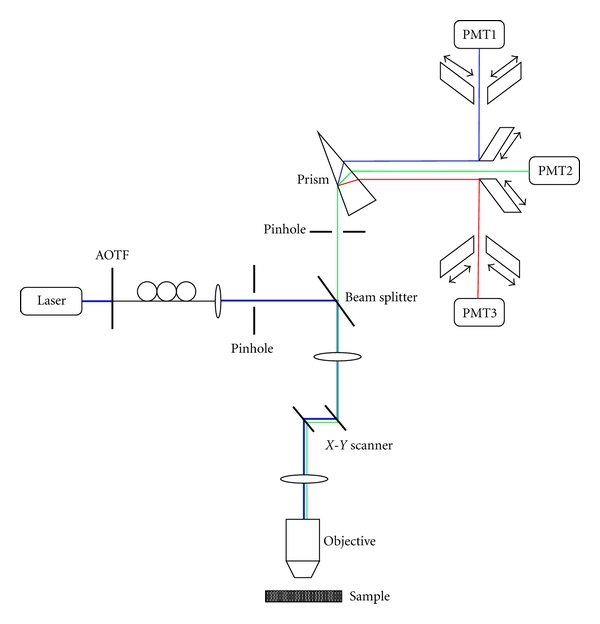
The schematic setup of our confocal microscope. The laser beam for excitation is incident upon the sample. With the prism, the signal splits into three wavelength ranges, each of which is detected by one PMT.

In our study, a confocal microscopic system (Leica TCS SP2) was used to obtain the multiwavelength fluorescence images of aorta samples. An Ar-Kr laser of 488 nm and a He-Ne laser of 543 nm are the excitation light sources.  Figure 1 shows the schematic setup of our confocal microscopic system. The laser beam passes though an acoustic optical tunable filter (AOTF) and is coupled into a fiber bundle. The AOTF is used to control the excitation intensity to avoid the photo-bleaching of the sample. The laser beam is then reflected to the sample by the dichroic beam splitter. The signal from sample is collected with an objective, passes though the dichroic beam splitter, and is finally incident on a prism. With the prism, the signal splits into three wavelength ranges, each of which is detected by one PMT [22]. The *X*-*Y* scanner is used to scan the sample for 2D imaging. The cut-open aorta tissue of a rat is prepared for *in vitro* measurement. The Ar-Kr laser excites the green autofluorescence from proteins in tissue, while the He-Ne laser stimulates the red extra-fluorescence from the cells of rat's endothelium labeled with dye alexa568 conjugated lectin GS-IB4. The scanning line rate is 400 Hz in the experiment.

The fluorescence images of rat's aorta segments were detected by a confocal microscope with the prism-based filter for the wavelength selection. In human bodies, the cholesterol cannot dissolve in blood. Lipoproteins play an important role in the cholesterol transport between cells. There are two kinds of lipoproteins: high-density lipoprotein (HDL) and low-density lipoprotein (LDL). HDL carries cholesterol away from the blood to prevent the heart disease. LDL, so-called “bad cholesterol”, builds up in the inner walls of the arteries. LDL causes the atherosclerosis when it combines with other substance and then induces the plaque generation in arteries [24, 25]. Thus, LDL indicates either leukocyte-endothelium interaction or platelet aggregation in the blood stream.

In this experiment, we observed the *in vitro* fluorescence images of a cut-open aorta of the rat [26, 27]. The relation between the VEcadherin and the activated leukocyte is verified experimentally. The samples are prepared under three distinct conditions: (1) the leukocyte is treated with 10 mg/mL native LDL solution and then mixed with the buffer-treated endothelial cells; (2) the leukocyte is treated with buffer and mixed with the endothelial cells, which are treated with 10 mg/mL native LDL solution; (3) the leukocyte and the endothelial cells are both treated with 10 mg/mL native LDL solution.

## 3. Results and Discussion

**Figure 2:**
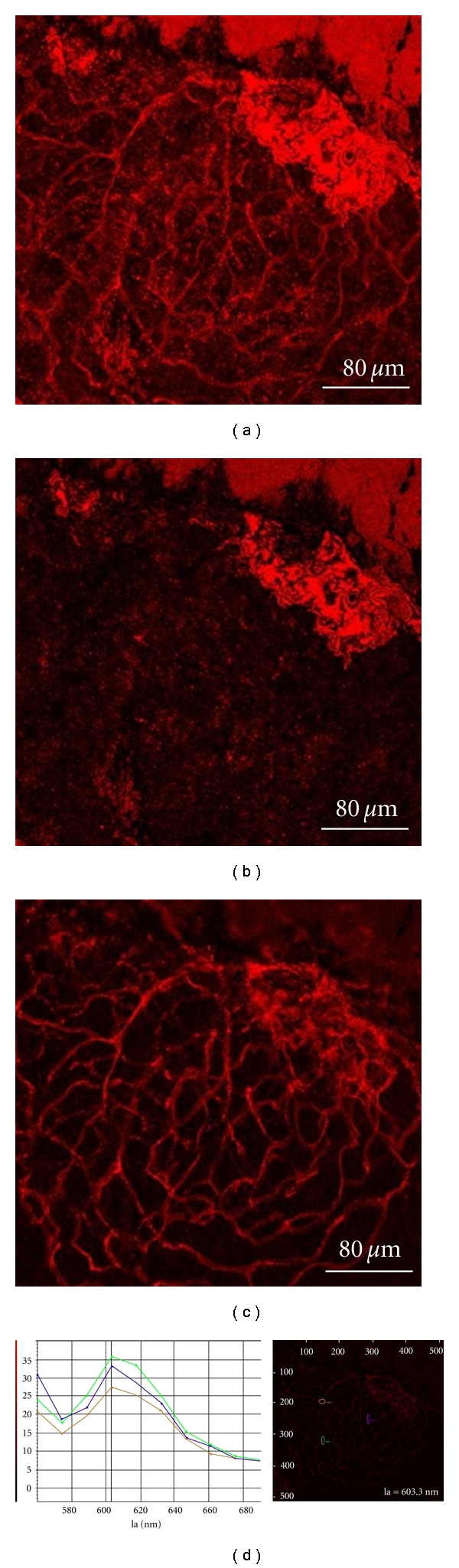
Red fluorescence images of the rat endothelial cells excited by He-Ne laser. The wavelength ranges are (a) 555–700 nm, (b) 555–575 nm, and (c) 600–630 nm, respectively. (d) The fluorescence spectra: each colored curve represents the data taken at the spot marked with the circle of that color.

The red fluorescence images of endothelial cells excited by He-Ne laser are shown in Figure 2.  Figure 2(a) is the image with the measured fluorescence range spanning from 555 nm to 700 nm, the TRITC fluorochrome range. In Figures 2(b) and 2(c), the measured wavelength ranges of fluorescence are 555–575 nm and 600–630 nm, respectively.  Figure 2(d) shows the fluorescence spectra of 3 selected spots, each marked with a distinct color. The spectral range is 560–700 nm at 20-nm wavelength intervals. The peak value of each fluorescence spectrum is about 605 nm. Obviously, Figure 2(d) shows the power of spectrum tail form incident He-Ne laser is larger than the fluorescence signal, thus it blurred the red fluorescence images. As a result, Figures 2(a) and 2(b) are the background images due to the backscattered photons of He-Ne laser. Therefore, we select optimal wavelength range in Figure 2(c) and it indicates the pure red fluorescence image from endothelial cells.

**Figure 3:**
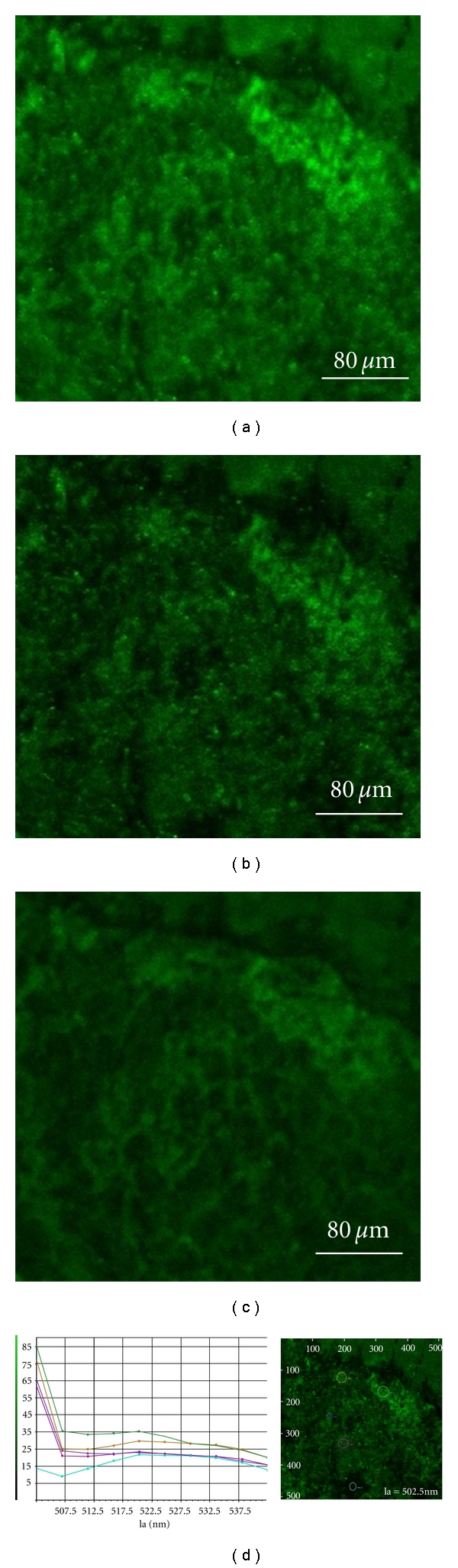
Green fluorescence images of the labeled protein excited by the Ar-Kr laser. The wavelength ranges are (a) 500–540 nm, (b) 500–505 nm, and (c) 515–535 nm, respectively. (d) The fluorescence spectra: each colored curve represents the data taken at the spot marked with the circle of that color.

The vascular endothelial cadherin (VECAD), the labeled protein, increases in number after the samples have been treated with LDL solution. The VECAD is an endothelial-specific cadherin localized at the intercellular junctions [28]. It could be associated with atherosclerotic lesions by endothelial cells and blood vessels forming [29]. The green fluorescence images from the labeled protein are shown in Figures 3(a)–3(c). The measured fluorescence range of Figure 3(a) is 500–540 nm, the FITC fluorochrome range. In Figures 3(b) and 3(c), the measured wavelength ranges are 500–505 nm and 515–535 nm, respectively. The fluorescence spectra with a wavelength range of 510–540 nm at 7.5-nm intervals are shown in Figure 3(d). The peak value of each spectrum is about 520 nm. Again, the spectrum tail from the Ar-Kr laser blurred the excited green fluorescence signal. Therefore, the 515–535 nm of measured wavelength ranges is selected and the real green fluorescence signal appears in Figure 3(c). The background signals due to the Ar-Kr laser fringes are shown in Figures 3(a) and 3(b).

The filter wheel with fixed wavelength range, for example, FITC, TRITC, and so forth, are not good enough for florescence imaging. In order to increase the image contrast as well as the signal-to-noise ratio and to avoid the spectral cross talk, an adjustable wavelength filter is a more appropriate option. Adopting the prism-based filter in the microscopic system could be a helpful tool to analyze fluorescence images immediately without the postprocessing or image reconstruction.

**Figure 4:**
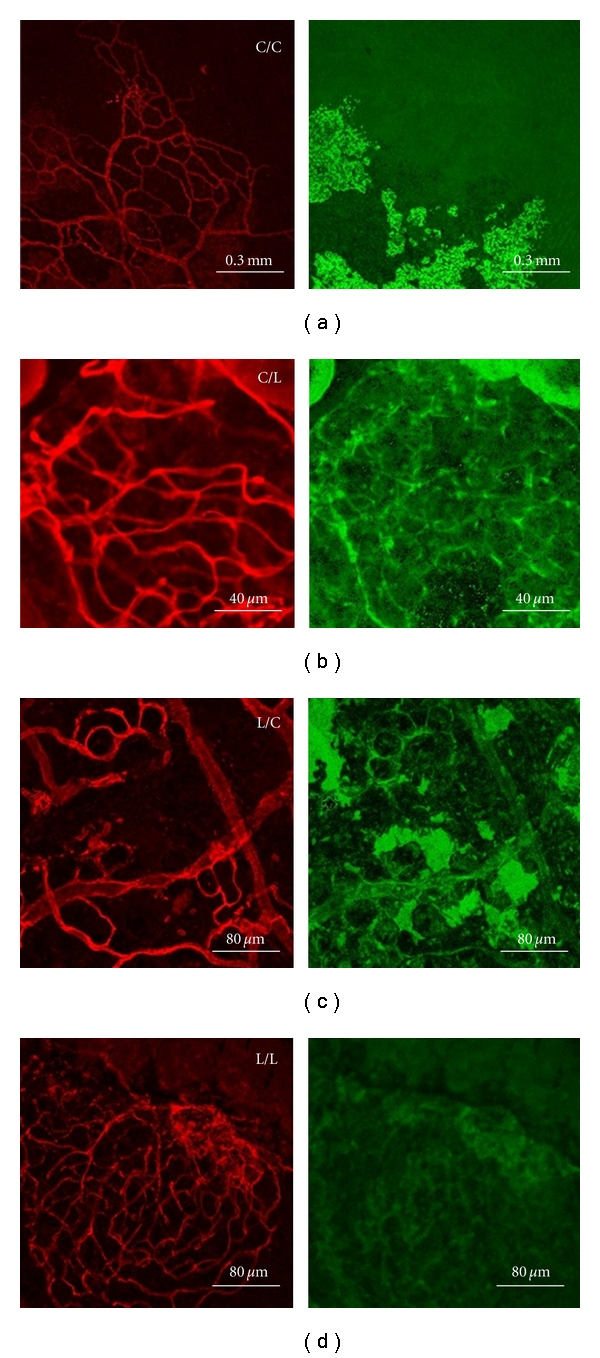
The comparison between red fluorescence images (left) excited by He-Ne laser and green fluorescence images (right) excited by Ar-Kr laser. (a) The leukocyte and the endothelial cells are both treated with buffer. (b) The leukocyte is treated with buffer and then mixed with the endothelial cells which are treated with 10 mg/mL native LDL solution. (c) The leukocyte is treated with 10 mg/mL native LDL solution and then mixed with the buffer-treated endothelial cells. (d) The leukocyte and the endothelial cells are both treated with 10 mg/mL native LDL solution.

Figure 4 shows the fluorescence images of the cut-open rat aorta treated with 10 mg/mL LDL solution and/or buffer solution. The wavelength ranges of the measured fluorescence are 600–630 and 515–535 nm for the red and green images, respectively. The first symbol of each label in Figure 4 stands for the treatment of leukocyte, and the second symbol represents that of the aorta segment. L stands for treatment with 10 mg/mL native LDL solution, while C means treatment with buffer solution.

In Figure 4(a), the leukocyte and endothelial cells do not treat with any extra solution. There is no green auto-fluorescence signal, which means no VECAD exists in the sample. In Figures 4(b)–4(d), with the leukocyte and/or rat's aorta segment treated with 10 mg/mL LDL solution, green autofluorescence is detected, meaning the increase in VECAD number. As mentioned before, the LDL solution activates the leukocyte to attack the rat's aorta endothelial cells and the VECAD number increases. There is then a rise of the possibility of atherosclerosis, which affects the arteries of the brain, heart, kidneys, vital organs, arms, and legs in human bodies

## 4. Conclusion

In this paper, we have demonstrated the measurement of multiwavelength fluorescence of the cut-open rat aorta using a Leica TCS SP2 confocal fluorescence microscope. The experimental results show that an adjustable wavelength filter is suitable for imaging optimization. The spectral range of 350–850 nm is wide enough for various kinds of fluorescence measurement. Using the Ar-Kr laser for green autofluorescence excitation and the He-Ne laser for red extra-fluorescence excitation achieves the multicolor images. The prism-based filter provides a flexible selection of wavelength pass range. The optimization of wavelength pass range suppresses noises from the excitation laser fringes, improving the contrast and signal-to-noise ratio.

In this experiment, the results show that the leukocytes attack the aorta endothelial cells after treatment with 10 mg/mL LDL solution, leading to the increase in number of VECAD. VECAD plays an important role in endothelial cell physiology. The cell contact regulates angiogenesis by controlling endothelial cell adhesion and migration and induces the generation of VECAD under a specific condition [30]. VECAD mediates the ability of leukocytes to go through the endothelial cells. Furthermore, it is considered to be associated with the dysfunction of endothelial cells and can accelerate atherosclerosis [31]. The confocal microscopic system is a useful tool for the measurements of biological properties with fluorescence detection of VECAD. It provides real-time imaging while the leukocytes are transporting through endothelial cells in the environment of tissue-culturing dishes and can observe the change of VECAD concentration immediately. Although our result shows only the *in vitro* confocal images, it still offers a good feasibility for cardiac endoscopy with fiber optics in clinical application.
